# “Neurological manifestations of COVID-19” - guideline of the German society of neurology

**DOI:** 10.1186/s42466-020-00097-7

**Published:** 2020-12-02

**Authors:** Peter Berlit, Julian Bösel, Georg Gahn, Stefan Isenmann, Sven G. Meuth, Christian H. Nolte, Marc Pawlitzki, Felix Rosenow, Benedikt Schoser, Götz Thomalla, Thomas Hummel

**Affiliations:** 1 Secretary General of the German Society of Neurology, Berlin, Germany; 2 Department of Neurology, Klinikum Kassel, DGNI, Kassel, Germany; 3 Department of Neurology, Klinikum Karlsruhe, DGNI, Karlsruhe, Germany; 4 Department of Neurology and Clinical Neurophysiology, St. Josef Hospital Moers, Moers, Germany; 5 Department of Neurology, University Hospital Düsseldorf, Düsseldorf,, Germany; 6 Department of Neurology with Experimental Neurology and Center for Stroke Research Berlin (CSB) Charité-University Berlin, Berlin, Germany; 7 Department of Neurology with Institute for Translational Neurology, University Hospital Münster, Münster, Germany; 8 Epilepsy Center Frankfurt Rhein-Main, Center of Neurology and Neurosurgery, University Hospital Frankfurt, Frankfurt, Germany; 9 Friedrich Baur Institute at the Neurological Department, LM-University Munich, Munich, Germany; 10 Department of Neurology, Head and Neurocenter, University Hospital Hamburg-Eppendorf, Hamburg, Germany; 11 Interdisciplinary Center for Smelling and Tasting, University ENT Hospital Dresden, German Society for ENT Medicine, Dresden, Germany

**Keywords:** SARS-CoV-2, COVID-19, Anosmia, Hyposmia, Encephalopathy, Encephalitis, Epileptic seizures, Seizure recurrence, Meningoencephalitis, Myelitis, Encephalomyelitis, Neuromuscular diseases, Myositis, Myasthenia gravis, Guillain-Barré syndrome, Miller Fisher syndrome, Critical illness weakness, Critical illness neuropathy/myopathy, Intensive care unit acquired weakness, ECMO, Ventilation, Delirium, Status epilepticus, Stroke, Intracerebral hemorrhage, Intracranial hemorrhage

## Abstract

Infection with the new severe acute respiratory syndrome coronavirus 2 (SARS-CoV-2) leads to a previously unknown clinical picture, which is known as COVID-19 (COrona VIrus Disease-2019) and was first described in the Hubei region of China. The SARS-CoV-2 pandemic has implications for all areas of medicine. It directly and indirectly affects the care of neurological diseases. SARS-CoV-2 infection may be associated with an increased incidence of neurological manifestations such as encephalopathy and encephalomyelitis, ischemic stroke and intracerebral hemorrhage, anosmia and neuromuscular diseases.

In October 2020, the German Society of Neurology (DGN, Deutsche Gesellschaft für Neurologie) published the first guideline on the neurological manifestations of the new infection. This S1 guideline provides guidance for the care of patients with SARS-CoV-2 infection regarding neurological manifestations, patients with neurological disease with and without SARS-CoV-2 infection, and for the protection of healthcare workers.

This is an abbreviated version of the guideline issued by the German Neurological society and published in the Guideline repository of the AWMF (Working Group of Scientific Medical Societies; Arbeitsgemeinschaft wissenschaftlicher Medizinischer Fachgesellschaften).

## Introduction

Infection with the new severe acute respiratory syndrome coronavirus 2 (SARS-CoV-2) leads to a previously unknown clinical picture, which is known as COVID-19 (COrona VIrus Disease-2019) and was first described in the Hubei region of China. The infection was declared a pandemic by the WHO on 11.03.2020. SARS-CoV-2 belongs to the community acquired respiratory viruses (CARV), which can cause upper and lower respiratory tract infections. The pathogen belongs to the group of coronaviruses, which can cause illnesses ranging from a normal cold to severe disease progression. Related to the SARS-CoV-2 virus are the viruses that cause the clinical picture of SARS (Severe Acute Respiratory Syndrome) and MERS (Middle East Respiratory Syndrome) and for which neurotropy has been demonstrated.

The SARS-CoV-2 pandemic has implications for all areas of medicine. It also directly and indirectly affects the care of neurological diseases. It is discussed that SARS-CoV-2 infection could be associated with the increased occurrence of neurological manifestations such as encephalopathy and encephalomyelitis, ischemic stroke and intracerebral hemorrhage, anosmia and neuromuscular diseases.

Many reports suggest a deterioration in the care of patients with neurological disorders due to the special demands on health care systems during the pandemic. Impacts on care can be seen directly and indirectly through the redistribution of resources in favor of SARS CoV-2 patients and protective measures for patients and caregivers. They affect the actions of laypersons (e.g. fear of infection in hospital), transport to hospital and intrahospital emergency care up to and including rehabilitation.

### The most important recommendations and statements of this guideline (all with approval rates between 90 and 100%)

#### Encephalopathy

- Encephalopathies are quite common in COVID-19, especially in severe cases.
- Symptoms and clinical course are highly heterogeneous.
- For encephalopathies triggered by SARS-CoV-2 are discussed as pathomechanisms: hypoxia, sepsis, severe systemic inflammation, renal failure and cytokine storm.
- Biomarkers found in this context in patients with severe COVID-19 were IL-2, IL-6, IL-7, GCSF, TNF-alpha1.
- A solid basis for specific therapeutic measures does not yet exist.

#### Meningoencephalitis

- In the case of newly occurring central neurological symptoms, especially in the case of disturbances of consciousness, acute cognitive deficits and epileptic seizures, further diagnosis with cerebral imaging (MRI), EEG examination and cerebrospinal fluid diagnostics is necessary.
- The use of corticosteroids in high doses may be attempted if symptoms persist.

#### Risk of COVID-19 under immunotherapy

An increased risk of COVID-19 disease under immunotherapy cannot be deduced from existing reports.

- It is advisable to continue immunotherapy. Individual risk factors such as patient age, morbidity and regional prevalence of COVID-19 should be taken into account to assess the individual patient risk and in individual cases de-escalation strategies such as a change of therapy or an extension of the interval should be evaluated.
- In case of COVID-19 disease, aspects such as disease activity of the underlying neurological disease as well as the previous course of therapy should be considered and the immune therapy should be paused if necessary.

#### Guillain-Barré syndrome (acute inflammatory demyelinating polyneuritis - AIDP)

- Guillain-Barré syndrome (GBS) is a serious complication of COVID-19 disease and can occur within days of the first respiratory symptoms.
- Clinically, mild courses up to severe tetraparesis and cranial nerve involvement are possible.
- Electroneurographically, a demyelinating pattern of damage usually dominates, although axonal processes are also reported.
- CSF diagnosis is necessary to exclude an infectious etiology. In most cases a cytoalbuminous dissociation appears.
- Serological testing of ganglioside antibodies is recommended.
- Intravenous immunoglobulins as well as plasma exchange procedures are to be regarded as equivalent and should be initiated promptly.

#### Acute disseminated encephalomyelitis

- Newly occurring multifocal neurological symptoms suggest acute disseminated encephalomyelitis (ADEM), so that rapid diagnosis including MRI and CSF analysis should be initiated.
- MRI imaging with contrast agent administration is essential for the detection of inflammatory lesions. A complementary hemorrhage-sensitive sequence helps to detect a hemorrhagic component.
- A normal CSF finding does not exclude the diagnosis of ADEM.
- A 3–5-day cycle with methylprednisolone 1 g/day should be administered intravenously. If symptoms persist intravenous immunoglobulins should be administered.

#### Stroke

- Worldwide, the number of patients hospitalized for stroke has fallen under the COVID 19 pandemic. In the pandemic, it is important for all physicians involved in stroke care to maintain the best possible quality of care for cerebrovascular diseases even under the current difficult conditions.
- Ischemic strokes and, more rarely, intracerebral hemorrhage (ICH) occur in patients with COVID-19 disease and are associated with a more severe course of the disease.
- When treating COVID-19 patients, it is important to detect possible cerebrovascular complications and immediately initiate the necessary diagnostic procedures.
- A confirmed or presumed infection with SARS-CoV-2 in patients with acute stroke should not lead to different treatment than for other stroke patients. They should receive the same acute diagnostics and acute treatment as all stroke patients, provided that appropriate hygiene measures are observed.

#### Epilepsy

- If epileptic seizures or a status epilepticus (SE) occur in patients with COVID-19 disease, it should be clarified whether it is a first-time seizure or a recurrence of previously known epilepsy.
- In case of unclear disturbance of consciousness, an EEG should be performed to detect and localize activity typical for epilepsy and to detect or exclude a non-convulsive SE.
- The treatment of seizures or SE should be performed according to the respective guidelines.
- Contraindications and interactions of anticonvulsants with substances used for COVID-19 disease should be taken into account.
- In patients with known fever-associated seizures, an NSAID (e.g. paracetamol) should be given.

#### Chemosensory disturbances

- Infection with SARS-CoV-2 can lead to chemosensory disorders, with hyposmia and often anosmia.
- During the pandemic, a suddenly appearing olfactory disorder (anosmia) during free nasal breathing is very likely an expression of an infection with SARS-CoV-2.
- Olfactory disturbances can precede other disease symptoms and are therefore epidemiologically relevant (early identification of new “hot spots”).
- The olfactory disorder in COVID-19 seems to be mostly temporary. Whether or not a complete restitution is regularly achieved cannot yet be conclusively assessed.
-Should the olfactory function does not return to normal within 3–4 weeks, a neurological and ENT presentation with further diagnostics is recommended.

#### Nerve and muscle affections

- Myalgia, fatigue and hyper-CK-emia are the most common triad forms (40–70%) of skeletal muscle affection in COVID-19 cohorts.
- A COVID-19 disease requiring intensive care with invasive ventilation can lead to ICUAW (“ICU-acquired weakness” [ICU: intensive care unit]), a clinical picture in which CIP (“critical illness polyneuropathy”) and CIM (“critical illness myopathy”) intertwine.
- There seems to be no massively increased risk for neuromuscular patients suffering from SARS-CoV-2 infection.

#### Neurological intensive care medicine

- Neurological manifestations of COVID-19 can easily remain masked in the severe, pulmonally dominated intensive care setting. Therefore, an active search for involvement of the central or peripheral nervous system is necessary.
- Invasive ventilation with PEEP (Positive End-Exspiratory Pressure), a permissive hypercapnia or in abdominal position can lead to an increase of the intracranial pressure, but still be necessary.
- If cerebral or spinal involvement by COVID-19 is suspected, a CT or MRI should be performed. In some patients who cannot be reliably examined clinically due to severe intensive care and/or analgosedation, this may also be indicated prophylactically (e.g. cerebral CT before ECMO).

### Neuroimmunological manifestations of COVID-19

#### (Infectious) inflammatory complications - (Meningo-)encephalitis

To date there are only a few case reports about the occurrence of a (meningo-)encephalitis in the context of COVID-19 [1–5]. It is unclear whether this is a direct SARS-CoV-2 infection of the CNS or an autoimmune post-infectious event. Taking into account previous experience with SARS-CoV-1, cerebral infection is possible, but rare [6]. Subacute appearance of neurological symptoms a few days after respiratory symptoms, which are often mild, indicates a directly infectious event. On the other hand, autoimmune encephalitis was reported after surviving pulmonary SARS-CoV-2 infections.

#### Diagnosis

In most cases, (sub-)acute cognitive deficits and impaired consciousness are dominating. (Non-)convulsive seizures or akinetic mutism may also be the initial symptom. Delayed weaning or persistent delirium after extubation should suggest a neurological involvement.

There are no specific MRI findings. Cortical hyperdensities with partial contrast uptake as well as extensive bilateral subcortical hyperdensities have been observed.

EEG-studies reveal unspecific changes, focal findings and epileptic discharges.

CSF findings range from normal cell findings to lymphocytic pleocytosis of > 100 cells/μl. A blood-cerebrospinal fluid barrier disturbance may be present. The direct detection of viral material by PCR is rarely successful. Standard pathogen diagnostics, especially for herpes viruses, should always be performed. In addition, autoantibody diagnosis from serum and cerebrospinal fluid is useful to exclude autoimmune encephalitis.

#### Therapy

In case of negative pathogen diagnostics and persistence of symptoms, high-dose therapy with methylprednisolone (1 g/day) may be attempted over a period of 3–5 days. There are also case reports for the subsequent use of plasma exchange procedures, although in these cases no infectious or inflammatory pattern could be detected by CSF diagnostics.

#### (Autoimmune) inflammatory diseases

##### Guillain-Barré syndrome (acute inflammatory demyelinating polyneuritis - AIDP)

There are several reports of Guillain-Barré Syndrome (GBS) in COVID-19 disease [2, 7–19]. As with other viral diseases, post-infectious genesis can be assumed, although the latency between the initial manifestation of COVID-19 and the occurrence of GBS appears to be very short.

##### Diagnosis

The neurological symptoms usually appear within 5–10 days after a COVID-19 diagnosis, although GBS may develop even weeks after infection. Due to the risk of cardiovascular complications, in particular respiratory insufficiency and cardiac arrhythmias, a rapid diagnosis and immediate therapy including critical care admission is recommended.

The symptoms range from mild sensitive deficits to severe tetraparesis. Cranial nerve involvement with bilateral facial nerve palsy, ocular muscle palsy or Miller Fisher syndrome are also reported. Often the rapidly progressive course leads to respiratory insufficiency and need of ventilation.

To date, no association between the severity of COVID-19 disease and the occurrence or course of GBS has been demonstrated. In some cases, the diagnosis of COVID-19 infection was made retrospectively. Consequently, a corresponding SARS-CoV-2 test is recommended with every new GBS diagnosis.

In CSF a “cytoalbuminary dissociation” with a total protein increase and normal or slightly increased cell count (0–10 cells/μl) is usually detectable. Intrathecal immunoglobulin synthesis and isolated oligoclonal bands are atypical. In addition, a serological test for ganglioside antibodies should be performed, particularly in cases of cranial nerve involvement.

##### Therapy

The therapy does not differ from the usual treatment for GBS. Up to now, the primary use of intravenous immunoglobulins (0.4 g/kg bw) is preferred, but plasma exchange is considered equivalent. Corticosteroids should be avoided.

##### Acute disseminated encephalomyelitis (ADEM)

ADEM occurs as a rare complication following an infection or vaccination and is usually characterized by a monophasic course. To date, there are few case reports of ADEM-like manifestations in the context of COVID-19 disease [12, 20–22]. It is striking that those affected so far have been in middle to old age.

##### Diagnosis

The clinical symptoms of ADEM vary significantly. Severe focal neurological deficits (optic neuritis, severe paresis) and a subacute encephalopathic syndrome may occur.

In MRI, large lesions in the medullary canal and basal ganglia, some of which absorb contrast medium, are typical. In addition, a blood-sensitive MRI sequence (T2* or SWI) should be performed to detect acute hemorrhagic leukoencephalitis [21, 22].

CSF testing usually reveals pleocytosis of < 100 cells/μl and sometimes a slight blood-brain barrier disturbance. Isolated oligoclonal bands in CSF are uncommon. Testing for aquaporin-4 or myelin oligodendrocyte glycoprotein (MOG) antibodies should be performed in order to identify the initial manifestation of a neuromyelitis optica spectrum disorder (NMOSD) or MOG encephalomyelitis.

##### Therapy

Therapeutically, the administration of high-dose corticosteroids (1–2 g/day) intravenously over 3–5 days with or without oral tapering is recommended. In case of an insufficient response to steroids, the administration of immunoglobulins (0.4 g/kg bw intravenously) is recommended.

##### General implications for immunotherapies in the time of the COVID-19 pandemic

Based on the few case reports of COVID-19 disease under immunotherapy, it is currently impossible to conclude whether immunotherapies are associated with an increased risk for COVID-19 or a poorer prognosis of the infection [23–28]. For multiple sclerosis (MS), however, epidemiological data from regional MS centers in Italy and Chile indicate a low incidence of COVID infections in these groups [29, 30]. In more than 2000 patients with NMOSD under appropriate immunotherapy in China, only two patients developed COVID 19 disease [31]. Table 1 provides an overview of currently applied immunotherapies for neurological disease patterns and recommendations in times of the COVID-19 pandemic and in case of acute COVID-19 disease.

**Table 1:** Overview of the most frequently applied immunotherapies for neurological diseases and corresponding recommendations regarding their application in times of COVID-19

Substance	Diseases	General Recommendation during pandemic	Specific recommendations in case of infection
***DNA-synthesis Interference strategies***			
**Azathioprin**	MG, NMOSD, PACNS, IIM, AIE, Vasculitis, Neurosarcoidosis	Continuation	Discontinuation
**Methotrexate**	MG, NMOSD, PACNS, IIM, AIE, Vasculitis, Neurosarcoidosis	Continuation	Discontinuation
**Cyclophosphamid**	PACNS, AIE, Vasculitis, Collagene vascular disease	Continuation, in case of longterm stable disease de-escalation	Discontinuation
**Mitoxantron**	SPMS	Alternative treatments, in case of longterm stable disease: dose-reduction or termination	Discontinuation
**Teriflunomid**	RRMS	Continuation	Continuation, in case of severe lymphopenia discontinuation
**Mycophenolat-Mofetil**	MG, NMOSD, PACNS, IIM, Vasculitis, Neurosarcoidosis	Continuation	Continuation, in case of severe lymphopenia discontinuation
**Cladribin**	RRMS	Delay next treatment cycle	Discontinue, look for alternate treatment
***Immune cell depletion strategies***			
**Rituximab**	MG, NMOSD, PACNS, IIM, AIE, CNS-Vasculitis, CIDP	Delay next treatment cycle; CD19-B-Zell-Monitoring	Discontinue, look for alternate treatment
**Ocrelizumab**	RRMS, PPMS	Delay next treatment cycle; CD19-B-Zell-Monitoring	Discontinue, look for alternate treatment
**Inebilizumab**	NMOSD	Delay next treatment cycle; CD19-B-Zell-Monitoring	Discontinue, look for alternate treatment
**Alemtuzumab**	RRMS	Delay next treatment cycle; CD19-B-Zell-Monitoring	Discontinue, look for alternate treatment
***Leucocyte sequestration***			
**Fingolimod/Ozanimod**	RRMS	Continuation	Continue, or discontinuation for a few weeks
**Siponimod**	SPMS	Continuation	Continue, or discontinuation for a few weeks
**Natalizumab**	RRMS	Continuation or delay of treatment cycle	Continuation or delay of treatment cycle
***Pleiotropic Immunomodulation***			
**Glatirameracetat**	RRMS	Continuation	Continuation
**Dimethylfumarat**	RRMS	Continuation Severe Lymphopenia: Discontinue	Continuation Severe Lymphopenia: Discontinue
***Cytokines***			
**IFN-β**	RRMS, SPMS	Continuation	Continuation
**Tocilizumab/Satralizumab**	NMOSD	Continuation	Continuation
***Complement inhibitors***			
**Eculizumab**	MG, NMOSD	Continuation	Continuation
***Intracellular pathway Blockers***			
**Ciclosporin A**	MG, IIM	Continuation	Continuation, dose reduction
***Acute therapies***			
**Steroid pulse therapy**	MS, MG, NMOSD, PACNS, IIM, AIE, Vasculitis, Neurosarcoidosis	Only in case of high disease activity	Discontinuation or dose reduction
**Chronic glucosteroid therapy**	NMOSD, MG, PACNS, IIM, CIDP, Vasculitis, Neurosarcoidosis	Dose reduction	Stable disease: Dose reduction
**IVIG**	MG, IIM, CIDP, GBS	Continuation or delay of treatment cycle	Continuation or delay of treatment cycle
**Plasmapheresis/Immuno-adsorption**	MS, MG, NMOSD, AIE, IIM, GBS	Continuation	Continuation

*AIE* autoimmune encephalitis, *GBS* Guillain-Barré syndrome, *CIDP* chronic inflammatory demyelinating polyneuropathy, *IFN-ß* interferon-beta, *IIM* idiopathic inflammatory myopathy, *IVIG* intravenous immunoglobulins, *MG* myasthenia gravis, *MS* multiple sclerosis, *NMOSD* neuromyelitis-optica spectrum disorder, *PACNS* primary angiitis of the central nervous system, *PPMS* primary chronic progressive multiple sclerosis, *RRMS* relapsing-remitting multiple sclerosis, *SPMS* secondary chronic progressive multiple sclerosis

### Acute encephalopathy and acute encephalitis associated with COVID-19

Encephalopathy is a vaguely defined, mostly reversible diffuse brain dysfunction without structural or direct infectious cause. Systemic infections can trigger septic encephalopathy or, in case of multi-organ failure, other types of metabolic encephalopathy.

Pathomechanisms for encephalopathies triggered by SARS-CoV-2 include sepsis, severe systemic inflammation, renal failure and cytokine storm.

Acute encephalitis may be caused by the direct infection of brain tissue with the virus. The virus may either directly damage brain cells through lytic replication cycles or through the cytotoxic immune response of the host organism. Often the meninges are also involved, which is why meningoencephalitis is usually a more appropriate term [31].

#### Diagnosis

The symptoms of encephalopathy and encephalitis include neuropsychological abnormalities, agitation and delirium, extrapyramidal-motor movement disorders, coordination disturbances, impairment of consciousness, epileptic seizures and focal neurological deficits.

COVID-19 cases with symptoms suggestive of encephalitis showed (sudden) olfactory and gustatory disturbances (10–70%), headaches (13%), dizziness (17%), hallucinations, confusion, dysexecutive disorders (after intensive care 36%), agitation (during intensive care 69%), vigilance reduction (8–15%), neuralgia (2%), epileptic seizures (1%), ataxia (1%), sudden neurological deficits (3%) or pyramidal tract signs (67%). Most cases were reported without CSF analysis, so that the presence of acute viral encephalitis cannot be assessed with certainty [12, 32].

Biomarkers found in patients with severe COVID-19 include IL-2, IL-6, IL-7, GSSF, TNF-alpha [1]. CT or MRI may detect structural lesions and brain edema. It may show focal brain edema and/or (multi-) focal contrast uptake or may also present hemorrhagic-necrotic changes. CSF analysis to exclude meningoencephalitis or to detect destructive markers after hypoxia is recommended. EEG should be used to monitor diffuse brain dysfunction and for the detection of (subclinical) epileptic seizures or status epilepticus. Triphasic waves may occur in hepatic or uremic encephalopathies.

#### Therapy

Symptomatic therapy aims at the control of the general homeostasis (electrolytes, fluid, temperature), neuroleptic or thymoleptic therapy of psychic elements, and anticonvulsive therapy of epileptic seizures. If the course of the disease is severe, supportive intensive care therapy is appropriate, including intubation and respiration, thrombosis prophylaxis, neuromonitoring, and escalating therapy of increased intracranial pressure.

### Cerebrovascular diseases

SARS-CoV-2 infection may be associated with an increased incidence of cerebrovascular diseases such as ischemic stroke and intracerebral hemorrhage. Numerous reports suggest that the care of patients with cerebrovascular diseases has deteriorated due to the special demands on health care systems during the pandemic. They affect the actions of laypersons (e.g. fear of infection in hospital), transport to hospital and intra-hospital emergency care up to and including rehabilitation.

#### SARS-CoV-2 as a risk factor for stroke

Several case series report rates of ischemic stroke in hospitalized COVID-19 patients ranging from 1.6 to 5%. Specifically, ischemic stroke rates were 3 in 184 (1.6%) in a Dutch case series [33], 9 in 362 (2.5%) in a case series from Milan, Italy [34], and 6 in 214 (2.8%) [35] and 11 in 221 (5%) [36] in two case series from Wuhan, China. The rate of cerebrovascular events was higher in patients with severe respiratory events. Patients with cerebrovascular events often had typical vascular risk factors. In a retrospective cohort study, patients with SARS-CoV-2 infection are reported to have a higher risk of acute ischemic stroke than patients with influenza infection [37]. A further analysis showed a rate of 32/3556 (0.9%) of strokes in hospitalized SARS-CoV-2 infected patients. However, stroke symptoms were the initial reason for admission in only 14/3556 (0.4%) (https://www.medrxiv.org/content/10.1101/2020.05.18.20105494v1).

Overall, the stroke rates in these case series thus seem to be in a range that is not unusual for patients with severe infectious diseases. These figures do not indicate a causally increased risk of stroke in patients infected with SARS-CoV-2. However, such an association is conceivable and as possible causes activation of the coagulation system, disseminated intravascular coagulation as well as vascular complications as an expression of severe organ damage are discussed. In a case series (*n* = 5) from New York, an accumulation of young stroke patients with large vessel occlusion treated by thrombectomy is reported [38]. Most of these patients did not show severe respiratory symptoms. Four of the 5 patients had delayed presentation of their stroke symptoms for hospital treatment.

The occurrence of intracerebral bleeding in patients with COVID-19 has also been reported [34], but here the data is even more limited and does not allow a reliable estimate of the frequency.

Patients with a history of cerebrovascular disease have a higher risk for a more severe course of COVID-19 disease. In a meta-analysis of the available studies on this topic, a stroke in the history was associated with a 2.5-fold increased risk of a severe course of the disease and a trend towards higher mortality [39].

#### Effects of the SARS-CoV-2 pandemic on the care of stroke patients

The COVID 19 pandemic has had an impact on the organization of stroke care worldwide. Two factors play an essential role here: Resources for acute care of stroke patients have been redistributed in many places in favor of the care of patients with COVID-19. Secondly, it can be observed that the number of stroke patients treated in hospitals has decreased significantly in many places during the COVID-19 pandemic. For example, in the Alsace region in France, in March 2020, 40% less stroke alerts, 41% less intravenous thrombolysis and 33% less thrombectomy treatments were observed compared to the previous year [40]. Similar figures were reported from Italy, where the number of hospitalized stroke patients decreased by 50% and thrombolysis patients by 25% [41]. China also reported a 40% decrease in hospital admissions for stroke and a 25% decrease in thrombolysis [42]. The main reasons for this are believed to be the uncertainty of the population and patients’ fear of infection during hospital treatment.

#### Diagnosis

In case of clinical signs and symptoms of stroke, immediate neurological consultation and appropriate diagnostic imaging by CT or MRI should be performed. It is advantageous to have a CT scanner specifically and exclusively for the diagnosis of patients with SARS-CoV-2 infection to minimize the possibility of infection. All these patients should be screened for SARS-CoV-2. The results of SARS-CoV-2 screening should be prioritized. Patients with SARS-CoV-2 infection, especially those with comorbidities, represent a risk group for the occurrence of cerebrovascular complications. In order to limit the risk of infection for the nursing staff, staff turnover should be limited (e. g. a permanent stroke COVID team is desirable). Hygiene standards should include appropriate protective clothing. Patients with COVID-19 should wear a mouth-nose cover, if tolerated. To further prevent transmission to other patients, they should be isolated. Ward areas for patients with cerebrovascular disease and COVID-19 can be designated and reserved for these patients. The care of patients with SARS-CoV-2 and cerebrovascular diseases must be coordinated on an interdisciplinary basis with all departments involved [43].

Patients with cerebrovascular diseases without (known) COVID-19 infection should be screened for SARS-CoV-2 to detect infection early and to take isolation measures. The use of telemedicine can reduce the risk of infection by reducing transport.

#### Therapy

Patients should receive acute treatment with intravenous thrombolysis or thrombectomy if indicated. Acute treatment of severe stroke must be carried out without interruption under protective measures. This applies in particular to thrombectomy in the cooperation of neurologists, interventional neuroradiologists, anesthetists and nurses due to the proximity to the patient and the risk of aerosol spread. Several professional societies entrusted with this setting have issued recommendations in this regard. These include the classification of each patient as suspected of COVID-19 triggering immediate testing, the preference for intubation anesthesia (to prevent possible uncontrolled emergency intubation during the intervention), video-laryngoscopic intubation, reduction of the number of people involved to the really necessary number and the use of personal protection and barrier material for patient and practitioner [44–49].

Patients who are hospitalized with acute stroke or intracerebral hemorrhage and who have been diagnosed or suspected to be infected with SARS-CoV-2 must be isolated immediately. The decision whether patients should be treated on a neurological stroke unit or on a ward designed for the care of patients with COVID-19 with appropriate monitoring facilities must be made on a case-by-case basis, depending on the hospital’s conditions (e. g. possibility of and experience with isolation of patients with SARS-CoV-2 infection on the stroke unit), and the patient’s clinical situation.

The COVID-19 pandemic and the associated protective measures for the population in general and in hospitals in particular must not lead to poorer care for stroke patients. Stroke remains to be a disease with often dramatic consequences and one of the main causes of permanent disability or death. While the health care system must be geared to the care of COVID-19 patients, it must also continue to ensure that the care of stroke patients is optimally organized. Hospitals must take appropriate organizational measures to ensure that they can continue to provide adequate care for patients with cerebrovascular diseases under the special conditions of the COVID-19 pandemic with the necessary protective and hygienic measures.

### Epileptic seizures and epilepsy in adults

#### Definition and classification

There are two clinical situations to be distinguished:

1. The first occurrence of seizures in COVID-19 patients and
2. The occurrence of COVID-19 disease in a patient with known epilepsy (prevalence 0.6–0.7% of the population)

Epileptic seizures during COVID-19 disease occur as acutely symptomatic seizures in the context of primary CNS involvement due to SARS-CoV-2 (meningoencephalitis, secondary CNS damage, e.g. COVID-19-associated stroke or ICH, and during ECMO therapy. Acute symptomatic seizures are defined as epileptic seizures occurring within 7 days of acute brain injury [50]. In the case of meningoencephalitis, this period may be longer, as the disease can remain active (acute) for a longer period of time. They also occur occasionally with pre-existing epilepsy of a different etiology. In the context of COVID-19 disease, seizure recurrences or -accumulations occur [51–53].

However, seizure occurrences are relatively rare. Lu et al. did not report acute symptomatic seizures in a cohort of 300 severely ill COVID-19 patients [54, 55]. In a further study of 214 COVID-19 patients (41% of whom were severely ill according to respiratory criteria), only in one patient (0.5%) a symptomatic seizure was observed [34]. Seizures may also occur as status epilepticus [56].

It is also known from the last SARS epidemic that patients with chronic epilepsy have reduced access to doctors and drugs and can therefore suffer drug withdrawal attacks [57]. For this reason, it is important to ensure that patients with pre-existing epilepsy always have access to outpatient neurological care and that sufficient and timely antiepileptic drugs are prescribed to prevent supply shortages [52].

#### Diagnosis

In case of an unclear disturbance of consciousness or in case of epileptic seizures, an EEG is needed. In patients with suspected COVID-19 disease, hyperventilation should not be performed to keep aerosol production low (personal protection) and to prevent destabilizing effects for the patients (patient protection).

EEG or EEG monitoring can also be used for therapy control. This is particularly useful in the case of a non-convulsive status.

Imaging is used to detect epileptogenic lesions and signs of increased intracranial pressure, e.g. in encephalitis, cerebral hemorrhage or acute ischemic stroke. In the acute situation, CCT is usually performed. If this is not sufficient, MRI is urgently recommended, as this method is more sensitive in detecting epileptogenic lesions and processes.

In addition to the general laboratory tests required for COVID-19, assessment of serum concentration of antiepileptic drugs (in the case of previously known epilepsy) is mandatory. Blood chemistry is also used to exclude other causes of acute symptomatic seizures such as electrolyte derailment or intoxication. If seizures occur as a result of COVID-19 infection, CSF studies are used to exclude or confirm meningoencephalitis as the cause.

#### Therapy

For acute symptomatic seizures and status epilepticus, antiepileptic therapy is carried out according to the two relevant DGN guidelines [58, 59]. For status epilepticus, benzodiazepines are usually given first. In the case of individual seizures followed by complete recovery, therapy with a rapidly and broadly effective antiepileptic drug is usually sufficient. In the course of antiepileptic therapy or in cases of pre-existing epilepsy with increasing seizures, the interaction of antiepileptic drugs with COVID-19 drugs should be taken into account [3]. A list of possible interactions can be found, for example, on the websites of the University of Liverpool (www.covid19-druginteractions.org).

### Disorders of the chemosensory system: anosmia, ageusia

#### Definition and classification

In the case of olfactory disorders, hyposmia and anosmia are differentiated, whereby complete anosmia denotes the complete loss of the olfactory capacity, whereas functional anosmia denotes a pronounced restriction of the olfactory capacity, in which a low residual perception, which is not relevant to everyday life, may still exist. Parosmias are changed perceptions of odors. Ageusia is a complete loss, while hypogeusia is a reduction of the sense of taste [60].

Odor disorders resulting from viral (flu) infections of the upper respiratory tract have long been known as postinfectious anosmia [61], and have been described after infections with a number of cold viruses, including adeno- and rhinoviruses [62–64].

In SARS-CoV-2, unlike many other respiratory viruses, the olfactory disorder is not predominantly associated with symptoms of rhinitis, so that direct damaging effects of the virus on the olfactory system are mainly discussed.

Corona viruses are neurotropic and can be neuroinvasive [63, 64]. An olfactory disorder in COVID-19 may reflect direct damage by SARS-CoV-2 at the olfactory epithelial or olfactory tract level [65] - or may indicate a more extensive invasion of the CNS via the olfactory tract into the CNS, as has been shown for other viruses [66]. SARS-CoV-2 has been detected in autopsies in the human brain [67]. Data on a possible longer-term persistence in the CNS and possible longer-term consequences in humans are not yet available.

#### Disturbances of the chemosensory system in COVID-19

For the first time, Mao et al. described neurological symptoms in COVID-19 patients, including olfactory disorders in 11/214 patients (5%) [68].

This was followed by a large number of reports on olfactory and taste disorders in Covid-19. The data are extremely heterogeneous, as preliminary and contradictory results were published very quickly during the pandemic. Even if details cannot be regarded as finally clarified, some essential points are currently emerging:

Smell and taste disorders are common with COVID-19. They appear especially in mild progressive disease, more often in previously healthy, in young people and in women more frequent than among men. Smell and taste disorders can be the first (and rarely the only) symptom of a COVID-19 disease.

Odor disorders in COVID-19 are often not associated with symptoms of rhinitis (sneezing, rhinorrhoea, congestion, obstruction) and differ in this respect phenomenologically and possibly also pathophysiologically from other post-viral olfactory disorders. Parosmias may occur during the course of the disease and the regeneration phase.

A new olfactory disorder/anosmia (with or without the subjective impression of an additional gustatory disorder) occurring during the pandemic should be an immediate cause for:

- Self-isolation/quarantine
- Testing for SARS-CoV-2 (via telephone contact with family doctor/health office)
- Use of personal protective equipment in professional contact with affected persons

#### Diagnosis

In the pandemic, sudden olfactory loss in patients without nasal obstruction has a specificity of 97% and a sensitivity of 65% for COVID-19 [69]. Due to the epidemiological importance for controlling the spread of SARS-CoV-2 during the pandemic, anamnesis, contact tracing, protective measures for contact persons are of particular importance. Exclusive self-disclosure regarding olfactory or taste disorders correlates only to a limited extent with objective findings [70]. However, due to the exposure risk for the investigator on the one hand and rather low individual relevance for the patients on the other hand, chemosensory testing is usually not used in acute situations, or disposable test systems that can be carried out independently are used, such as the UPSIT test for olfactory identification [71, 72]. If necessary (e.g. for epidemiological reasons and in situations where SARS-CoV-2 cannot be tested directly by throat swab and RT-PCR), self-testing of patients in domestic quarantine with household fragrances is possible [73, 74].

There are several reports of either mucosal swelling with secretion in the olfactory duct (CT, MRI) or signal changes in the olfactory bulb and/or nerve (MRI). In individual cases, changes in the orbitofrontal cortex have also been described. The relevance of these findings cannot yet be conclusively assessed.

#### Course and therapy

The course of olfactory and taste disorders with COVID-19 is generally favorable: a majority of patients report complete or extensive improvement within 2–3 weeks. In about 10–20% of the cases relevant limitations remain. Thus, the prognosis is probably more favorable than for post-viral olfactory disorders of other etiologies. A survey described a good improvement within 1 year in 80% of the patients after a postviral olfactory disorder (without further differentiation of etiology) [75]. In other studies, in which olfactory ability was measured, an improvement of about 30% was reported for postviral olfactory disorders in about the same period [76].

If an olfactory disorder in the context of COVID-19 disease has not largely regressed within 4 weeks, a neurological and ENT examination should be performed, including a medical history (including competing/alternative causes) and examination, usually after a negative throat swab. This includes an olfactory and taste test, supplemented by laboratory diagnostics, imaging and endoscopy. If an olfactory disorder persists for a longer period of time, a therapy with consistent, structured “olfactory training” can be attempted. Classically, the following are used for this purpose: rose, lemon, eucalyptus [77]..

### Neuromuscular diseases

The combination of myalgia, fatigue and elevation of blood-creatine kinase levels is the most common form (40–70%) of skeletal muscle affection in COVID-19 cohorts [34, 78–80]. Rhabdomyolysis was detected in 0.2% and elevated CK levels in 13.7% in a series of 1099 COVID-19 patients [79, 81]. In up to 30% of patients, the increase of CK was found after 1–2 days. Only one case report has been published regarding the occurrence of a myositis associated with a SARS-CoV-2 infection [82]. In Italy, three patients with initial manifestation of acetylcholine receptor antibody-positive myasthenia gravis were observed 5 to 7 days after the onset of febrile SARS-CoV-2 infection [83].

Deterioration of a pre-existing neuromuscular disease has been described for amyotrophic lateral sclerosis (ALS) and myasthenia gravis [84].

#### Peripheral nervous system

Case reports and smaller case series of SARS-CoV-2-triggered Guillain-Barré syndrome (GBS) have been published [85, 86]. A GBS can develop within 5–10 days after the onset of COVID-19 symptoms. Additionally, few patients with Miller Fisher syndrome (MFS) have also been reported [87]. So far, there is no evidence of chronic inflammatory demyelinating polyneuropathy (CIDP) in relation to SARS-CoV-2 [84].

COVID-19 patients requiring intensive care with invasive ventilation may develop ICUAW (“ICU-acquired weakness” [ICU: intensive care unit]), a syndrome in which CIP (“critical illness polyneuropathy”) and CIM (“critical illness myopathy”) intertwine [88, 89]. The incidence of ICUAW increases with the severity and duration of the intensive care treatment and is compounded by sepsis, multiorgan failure, hyperglycemia, parenteral nutrition and certain medications (sedatives, antibiotics, corticosteroids, muscle relaxants). This clinical picture is characterized by weakness and atrophy of the entire musculature, including the respiratory muscles, and sensory disturbances.

#### Diagnosis

In outpatients, the standard diagnostics of neuromuscular diseases (history taking, clinical examination, EMG/NLG, pulmonary testing, creatine kinase assessment, and, in some cases, CSF chemistry should be performed under appropriate hygiene measures. In intensive care units, a bedside EMG can also be carried out in compliance with hygiene measures. MRI of the musculature is rarely indicated in the risk-benefit analysis due to the high effort involved, combined with an increased patient risk in patients requiring intensive care and the necessary final disinfection of the MRI machine. If an imaging examination is urgently required, a muscle sonography can be performed. Muscle biopsies may be indicated in rare cases.

#### Therapy

The therapy for inflammatory/autoimmune-associated diseases of the musculature, the neuromuscular junction, and the peripheral nerve should follow the current guidelines, including therapeutic measures such as plasmapheresis and immunoglobulins. Symptomatic treatment (e. g. pyridostigmine and 3,4-diaminopyridine/ampiridine) and immunomodulatory therapy (eculizumab) may be continued in consideration of the individual benefit-risk profile. The administration of rituximab or the initiation of oral long-term immunosuppression should be delayed depending on the clinical condition of the patient and the patient’s medical history [90, 91].

Vaccination recommendation for healthy individuals in the same age groups (influenza and pneumococcal vaccination) apply also for neuromuscular patients.

#### General recommendations in case of respiratory deterioration of ventilated patients

In case of respiratory decompensation of ventilated patients or in case of newly occurring respiratory impairment after exposure to COVID-19, telephone or telemedicine contact with the treating physician and the respiratory care provider is recommended.

In Germany, neuromuscular patients are managed in neuromuscular centers, by telephone and video or as outpatients and inpatients while maintaining the necessary distance. There is a close exchange with patient organizations such as the German Society for Muscle Patients and the World Muscle Society (WMS). Specific prevention and therapy recommendations have been published [91, 92]. Specific measures for patient transport and the hospital are the use of masks with a bacteria-virus filter at the device outlet and a filter between mask and device hose. It is important for physicians in emergency rooms and intensive care units to consult neuromuscular specialists and pneumologists when deciding whether to admit, escalate or terminate treatment of patients with neuromuscular diseases and COVID-19.

So far, due to the adherence to general hygiene regulations with self-isolation measures, only very few neuromuscular patients in Germany have been infected with SARS-CoV-2. Thus, there does not seem to be a massively increased risk for neuromuscular patients undergoing COVID-19 infection.

### Neurological critical care medicine

Reports of severe neurological involvement such as encephalitis, encephalopathy, status epilepticus, ischemic and hemorrhagic strokes and severe neuropathies (Guillain-Barré syndrome) in COVID-19 are increasing, which makes this problem particularly relevant to neurological critical care therapy.

The nervous system can be directly or, more frequently, indirectly be involved. This distinction is important for diagnostic and therapeutic decisions, and the prognosis of COVID-19 patients. The knowledge of neurological involvement may also play a role in the strategy of supportive intensive care therapy. For example, some forms of therapy such as ECMO could be unfavorable in the presence of extensive brain infarctions. Neurological manifestations of COVID-19 can easily remain masked in the severe, pulmonary dominated intensive care situation. Therefore, an active search for involvement of the central or peripheral nervous system is necessary.

The general principles of intensive care for COVID-19, are summarized in the guideline “Recommendations for intensive care therapy of patients with COVID-19” of the DGIN and DIVI [93, 94].

Neurological manifestations of great importance for intensive care medicine are encephalopathies, meningoencephalitis, severe ischemic or hemorrhagic strokes, rapidly progressing polyneuropathies and intensive care unit-acquired weakness.

In addition to the usual diagnostics for SARS-CoV-2, a RT-PCR test from CSF should also be performed if clinical suspicion of encephalitis, delirium or polyneuritis exists and there are no contraindications against lumbar puncture.

An EEG should be performed if the wake-up reaction is unclear, a delirium exists, or if there are indications of convulsive or nonconvulsive status epilepticus.

In the case of focal neurological symptoms cerebral and/or spinal MRI imaging should be performed. In some patients who cannot be reliably examined clinically due the intensive care situation and/or analgosedation, this may also be indicated prophylactically (e.g. cerebral CT before ECMO in order to exclude hemorrhagic or ischemic stroke) [38, 95].

Optimization of ventilation parameters can influence intracranial pressure. Invasive ventilation with PEEP (Positive End-Exspiratory Pressure), permissive hypercapnia or in prone position can lead to an increase of intracranial pressure. However, the data on this is inconsistent. Therefore, the procedure always requires an individual risk-benefit assessment [96–98].

Multimodal neuromonitoring (e.g., ICP/CPP measurement, NIRS, transcranial Doppler/Duplex, sonographic measurement of the optic nerve sheath diameter) can facilitate the therapeutic procedure in patients with increased intracranial pressure [99].

## Data Availability

Not applicable.
